# Supplementary material for: A UMLS-based spell checker for natural language processing in vaccine safety
Source: BMC Med Inform Decis Mak. 2007 Feb 12;7:3. doi: 10.1186/1472-6947-7-3 (PMC1805499; doi:10.1186/1472-6947-7-3)
Supplement: Additional file 13 — RAP application directory. Sets up directory for RAP (RDF application for PHP) source code [file 1472-6947-7-3-S13.gz › rap/doc/phpdoc/__filesource/fsource_infModel__infModelInfStatement.php.html]

xml version="1.0" encoding="iso-8859-1"?


File Source for InfStatement.php


# Source for file InfStatement.php

Documentation is available at InfStatement.php

```
1. <?php
2. // ----------------------------------------------------------------------------------
3. // Class: InfStatement
4. // ----------------------------------------------------------------------------------


8. /**
9. * An RDF statement which was entailed by a inference rule.
10. * See Statement Class for further information.
11. *
12. * <BR><BR>History:<UL>
13. * <LI>09-07-2004                : First version of this class.</LI>
14. *
15. * @author Daniel Westphal <mail at d-westphal dot de>
16. * @version  V0.9.1
17. * @package infModel
18. */
19. class InfStatement extends Statement  {

21. }

23. ?>
```

Documentation generated on Thu, 7 Jul 2005 13:41:45 +0200 by phpDocumentor 1.3.0RC3
